# Financial Wellbeing and Quality of Life Among a Sample of the Lebanese Population: The Mediating Effect of Food Insecurity

**DOI:** 10.3389/fnut.2022.906646

**Published:** 2022-07-26

**Authors:** Joanne Karam, Chadia Haddad, Hala Sacre, Mireille Serhan, Pascale Salameh, Lamis Jomaa

**Affiliations:** ^1^Nutrition Department, Institut National de Santé Publique, d'Épidémiologie Clinique et de Toxicologie (INSPECT-LB), Beirut, Lebanon; ^2^School of Health Sciences, Modern University of Business and Science, Beirut, Lebanon; ^3^Research Department, Psychiatric Hospital of the Cross, Beirut, Lebanon; ^4^Department of Nutritional Sciences, Faculty of Health Sciences, University of Balamand, Koura, Lebanon; ^5^Department of Primary Care and Population Health, University of Nicosia Medical School, Nicosia, Cyprus; ^6^School of Medicine, Lebanese American University, Byblos, Lebanon; ^7^Faculty of Pharmacy, Lebanese University, Hadat, Lebanon; ^8^Department of Human Sciences, College of Health and Sciences, North Carolina Central University, Durham, NC, United States; ^9^Department of Nutrition and Food Sciences, Faculty of Agricultural and Food Sciences, American University of Beirut, Beirut, Lebanon

**Keywords:** food insecurity, quality of life, financial wellbeing, Lebanon, physical health, mental health

## Abstract

**Background:**

Lebanon is undergoing multiple overlapping crises, affecting the food security, financial well-being, and quality of life (QOL) of its residents.

**Objective:**

The primary objective was to assess the food insecurity (FI) status of a sample of the Lebanese population. The second objective was to explore factors related to QOL parameters and evaluate the mediating effect of food security between financial well-being and QOL.

**Methods:**

The study was cross-sectional and enrolled 412 participants recruited online using the snowball sampling technique. The survey included questions related to sociodemographic and economic characteristics of Lebanese households and validated scales to assess FI, QOL measures, financial well-being, and fear of COVID-19.

**Results:**

Almost 43% of the study participants reported being food insecure, with 31% experiencing mild FI, 10% moderate FI, and 1.5% severe FI. Compared to food-insecure participants, food secure participants had a significantly higher income (58.5% vs. 39.2%, *p* < 0.001), a university education level (96.6% vs. 88.1%, *p* = 0.002), an average perceived financial status (83.9% vs. 65.9%), higher financial well-being scores (5.14 vs. 3.19, *p* < 0.001), and lower crowding index (0.94 ± 0.4 vs. 1.09, *p* = 0.002). Multivariate analysis showed that FI was not associated with physical (Beta = −1.48, 95% CI: −3.10; 0.13) and mental (Beta = −1.46, 95% CI −3.68; 0.75) QOL, after adjusting for other demographic and socioeconomic correlates. This association remained non-significant when introducing the financial well-being variable to the model. Mediation analyses showed that the FI variable mediated the association between financial well-being and physical QOL (Beta = 0.19, 95% CI: 0.02; 0.36), but not the mental QOL (Beta = −0.02, 95% CI: −0.20; 0.14).

**Conclusion:**

Food insecurity was prevalent in our study sample, and it mediated the association between financial well-being and physical, but not mental, QOL parameters. These findings call for evidence-based policies and programs to help improve the food security and well-being of Lebanese households amidst these unprecedented circumstances.

## Introduction

Lebanon once considered the Switzerland of the Levant, and its capital, Beirut, the “Paris of the Middle East” (1), are facing an escalating humanitarian juncture arising due to multiple concurrent crises: a massive economic collapse, the August 4 tragic Beirut Port blast, and the ongoing COVID-19 pandemic (2). These overlapping crises continue to have serious repercussions on the country's economic viability, political stability, and the food and health security of its population.

According to a report published by the World Bank (3), the economic and financial hardship that Lebanon has been undergoing in the last 2 years was ranked among the top three most severe crises globally since the mid-nineteenth century. The currency in Lebanon lost its value in a dramatic way, the income of 1,500,000 LBP (Lebanese Pounds) was worth 1,000 USD prior to the crises, and have declined significantly reaching 60 USD at the time when paper was released. In parallel, people have limited access to their savings in the banks and can retrieve from it small monthly amount in foreign currencies. This left the population in Lebanon with financial instability and inability to cope with emergencies. Alongside the economic crisis, unprecedented price inflation for all commodities, including food and beverages, reached up to 483% in January 2022, after being 438.65% in December 2021, according to the Central Administration of Statistics (4). In 2020, the United Nations estimated that over 50% of the Lebanese population was at risk of failing to access basic food needs (5). The collapse of the financial system, the weak welfare programs, and the ever-increasing economic, social, and political challenges facing Lebanon since 2019, have all threatened the food security and health of its residents (6, 7).

Food insecurity (FI), defined by the Food and Agriculture Organization (FAO) as the lack of physical, economic, and social access to safe, sufficient, and nutritious food (8), affects physical and mental health, both considered dimensions of quality of life (QOL), along with social health, material wellbeing, and development and activity (9, 10). FI has been associated with multiple nutrition-related health outcomes, including, but not restricted to, dietary inadequacies, obesity, poor general health, and a myriad of chronic health conditions among adults (11, 12). In addition to its physical health effects, FI has also been associated with poor mental health outcomes related to its degree, with severe FI being associated with extreme chronic stress (13) and a higher risk of anxiety and depression (14). Multiple potential mechanisms can explain the association of FI and poor mental health, such as the increased physical stress caused by poor nutrition and nutrient insufficiency, the psychological stress caused by the anxiety of not obtaining enough food, or the social stress, including isolation and shaming (15).

Scientific evidence published since the start of COVID-19 highlighted the myriad of adverse effects of the pandemic on health outcomes, including increased psychological stressors and poor mental health outcomes. Studies showed that populations globally were undergoing panic, fear, and phobia that extended beyond the fear of being infected or infecting others to anxiety about loss of jobs, lower incomes, and lack of access to food and other basic amenities. This issue was particularly pronounced among vulnerable populations residing in low-to-middle-income countries (LMICs) with limited social protection programs (14, 16–18).

In Lebanon, the COVID-19 pandemic coupled with economic and financial hardships, and political instability, has adversely impacted the food security of the population and their physical and mental health (19, 20). Recent studies have highlighted the compromised mental health of Lebanese, an essential pillar of quality of life (QOL) (20), reflected by increased incidences of post-traumatic stress disorders and depression symptoms following the Beirut blast (16, 20). A recent Lebanese study conducted between November and December 2020 among 1,133 Lebanese participants found that food insecurity is an immediate problem for households in Beirut and many governorates in Lebanon (19). Another recent study that aimed to evaluate the impact of the pandemic and economic crises on FI in Lebanon revealed that FI was estimated to reach 36 to 39% post-crises, on average (21).

Theoretically, it is known that financial constraint is related to FI (22) and it is an indicator of the quality of life and wellbeing (23). In turn, FI was found in the scientific literature to be related to physical (11, 12) and poor mental health (13, 14). However, in the absence of a framework, exploring the relationships between these factors (financial wellbeing, FI and QOL), it was interesting to study these associations. Also, to our knowledge, no study showcased the association of FI with the QOL parameters among the Lebanese population and none have explored if FI in Lebanon, amidst the multiple crises, was directly affecting mental (wellbeing, anxiety, depression) and physical QOL, or if QOL parameters are affected by other underlying causes including, but not restricted, to past traumas, fear of COVID-19, and fear of poverty. Therefore, this study aimed to present a better understanding of the association between these variables that affect to a great extent the Lebanese population. The primary objective of the current study was to assess the food security status of a sample of the Lebanese population amidst multiple overlapping crises in the country. The second objective was to explore factors related to physical and mental QOL and to evaluate the mediating effect of food security between financial wellbeing and QOL.

## Materials and Methods

### Study Design and Population

This study was cross-sectional and enrolled 412 Lebanese adults from all governorates recruited online between October and December 2021, using the snowball sampling technique, which is a non-probability sampling technique. Selected participants were asked to enroll future subjects for the study by sharing the questionnaire with their peers and contacts via social media sites. An anonymous self-administered Arabic questionnaire was developed and shared by the researchers on several social media platforms (WhatsApp, Facebook, Instagram, and Linkedin). Non Lebanese living in Lebanon, Lebanese living abroad and participants younger than 18 years old were excluded from the study.

### Ethics Approval

The study was conducted according to the guidelines laid down in the Declaration of Helsinki, and all procedures were approved by the Modern University of Business and Science Ethics Committee (approval reference MU-20211005-26, Oct 2021).

### Sample Size Calculation

The Epi Info™ software (Centers for Disease Control and Prevention, Epi Info™) was used to calculate the minimum sample size. Considering a prevalence of 36% of individuals having food insecurity based on a recent Lebanese study done by Kharroubi et al. (21), with a 95% confidence level and an alpha error of 5%, the sample size needed was 354 participants. The final sample included 412 participants to take into account non-response or missing data. The sample size was determined before initiating the study.

### Questionnaire

The online survey tool was in Arabic language and comprised of two sections. The first part of the questionnaire consisted of sociodemographic and economic status and other descriptive characteristics of the participants. The second part included validated scales to assess food security, quality of life (QOL) measures, financial wellbeing, and fear of COVID-19.

#### Sociodemographic, Economic Status and Other Descriptive Characteristics of the Study Participants

Sociodemographic status included questions on gender (male, female), age (in years), marital status (single, widowed, not married, married), educational level (primary/below 5 years of age), intermediate/below 12 years of age, secondary/below 18 years of age, and university), employment status (employed, unemployed), region of residence (governorates: Beirut, Mount Lebanon, South, North, Beqaa), area of residence (city, village), and household crowding index. The latter was calculated by dividing the number of persons living in the house by the number of rooms, excluding the bathrooms and kitchen. Economic status included questions on monthly income/financial status divided into no income, low (<1,000 USD), intermediate (1,000-2,000 USD), high (>2,000 USD), and refuse to answer. The income of 1,500,000 LBP (Lebanese Pounds) was worth 1,000 USD prior to the crises. Currently, it is worth 60 USD.

Moreover, fear of poverty was measured on a Likert scale from 0 to 10, where zero indicates no fear of poverty and ten extreme fear of poverty. Also, questions with three options of answer (yes, no, refuse to answer) were asked about the source of income at home, whether it is from work, aids from relatives, aids from governmental or non-governmental institutions, or income/aid in foreign currency. Other characteristics were also explored: taking medications for insomnia or depression or anxiety, alcohol consumption, and smoking status.

#### The Food Insecurity Experience Scale

Food insecurity was assessed using the validated Arabic version of the Food Insecurity Experience Scale (FIES), an experience-based measure of food insecurity developed by the Food and Agriculture Organization, Voices of the Hungry project (24). FIES illustrates people's experiences in accessing food in the past twelve months using an 8-item scale investigating the ability to obtain enough food, households running out of food, and being forced to compromise the quality or quantity of food due to limited financial resources of respondents. For each of the eight questions of FIES, responses were coded as Yes (=1) and No/I don't know/I don't want to answer (=0). The sum of the eight responses was then calculated to obtain the raw score of FIES per household. A raw score of 0 indicated food security, while higher raw scores above 0 indicated FI, divided as follows: mild FI (1-3), moderate FI (4-6), and severe FI (7-8). In addition a binary variable (food secure vs. food insecure) was created to display those having or not FI. In this study, the Cronbach alpha value was 0.806.

#### The 12-Item Short-Form Health Survey (SF-12)

Health-related quality of life was assessed using the validated Arabic version of the 12-item short-form health survey (SF-12) (25). This scale was developed by the World Health Organization (WHO) and provided an overview of mental and physical functioning and overall health-related QOL. The survey derived two summary scores: physical and mental component summaries (PCS and MCS). PCS and MCS range from 0 to 100, where 0 indicates the lowest level of health measured by the scales and 100 indicates the highest level of health (26). In this study, the Cronbach alpha value of the PCS was 0.466 and 0.454 for the MCS.

#### The InCharge Financial Distress/Financial Wellbeing Scale

Financial wellbeing was assessed using the validated Arabic version of the InCharge Financial Distress/Financial Wellbeing (IFDFW) scale, an 8-item self-reported measure of perceived financial distress/financial wellbeing, representing responses to one's financial state on a continuum ranging from overwhelming financial distress/lowest level of financial wellbeing to no financial distress/highest level of financial wellbeing (27). In this study, the Cronbach alpha value was 0.924.

#### The Fear of COVID-19 Scale (FCV-19S)

The validated Arabic version of the Fear of COVID-19 Scale (FCV-19S) (28) is a 7-item scale designed to assess anxiety and fear related to the COVID-19 pandemic. Examples of questions included “I am most afraid of Corona,” “It makes me uncomfortable to think about Corona,” “My hands become clammy when I think about Corona.” The items are rated on a 5-point Likert scale ranging from 1 (strongly disagree) to 5 (strongly agree). A total score could be calculated by summing each item score (ranged from 7 to 35) (29). A higher score indicates a greater fear of COVID-19. In this study, the Cronbach alpha value was 0.912.

### Statistical Analysis

The SPSS software version 25 was used to analyze data. Descriptive analyses were performed, where categorical variables were expressed as absolute frequencies and percentages and continuous variables as means and standard deviations. In order to describe the level of FI the ordinal categories of FI scale were used. The sample was normally distributed as verified by the visual inspection of the histogram, while the skewness and kurtosis were below |1.96|. Also, the normality of the MCS and PCS scales was verified by the normality line of the regression plot and the scatter plot of the regression residuals. The independent-sample *t*-test was used to compare continuous variables between groups, whereas the ANOVA test was used to compare between three or more means. For the comparison between categorical variables, the chi-square test was used. Pearson correlation test was used to evaluate the association between continuous variables. Cronbach's alpha values were recorded for reliability analysis for all the scales used in the present study.

A logistic regression model was conducted to explore the correlates of food insecurity, taking the binary variable of FI (food secure vs. food insecure) as the dependent variable. In addition, a three-stage linear regression analysis was performed, taking the two components of quality of life (PCS-SF12 and MCS-SF 12) as the dependent variables. In model 1, the relation between sociodemographic variables and QOL parameters (PCS-SF12 and MCS-SF12) was explored. In model 2, the binary food security variable was added to the analysis. In model 3, the relationship between food security, financial wellbeing, fear of COVID-19, alcohol consumption, and smoking status with QOL parameters was explored, adjusted for sociodemographic variables (Gender, age, household crowding index, education level, financial status, fear of poverty, monthly income and household crowding index).

**Figure 1:**
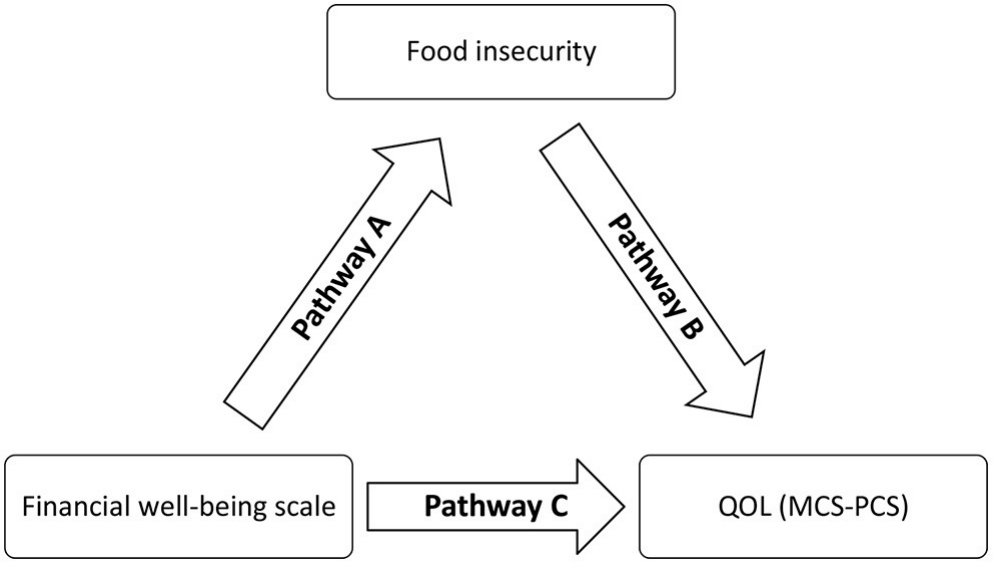
The framework showing the mediation pathways between financial wellbeing, food insecurity, and quality of life.

Moreover, the PROCESS SPSS Macro version 3.4 model four was used to calculate three pathways in the mediation analysis. The FI variable was considered as a continuous variable and entered as a mediating variable in the model. Pathway A determined the regression coefficient for the effect of financial wellbeing on food security. Pathway B examined the association of food security with PCS-SF12 and MCS-SF12, and pathway C estimated the total and direct effect of the financial wellbeing scale on QOL (MCS and PCS) (Figure 1). The macro generated bias-corrected bootstrapped 95% confidence intervals (CI) to test the significance of the indirect effect. Mediation was significant when the CI around the indirect effect did not include zero. The covariates that were included in the mediation model were those that showed significant associations with the PCS and MCS scales in the bivariate analysis. A *p*-value < 0.05 was considered statistically significant.

## Results

### Sample Characteristics

Table 1 shows the sociodemographic and other characteristics of the participants. Slightly less than half of the study participants (47.3%, *n* = 176) reported being food insecure with 31% (*n* = 128) experiencing mild food insecurity, 10% (*n* = 41) moderate food insecurity, and 1.5% (*n* = 6) severe food insecurity.

**Table 1:** Socio-demographic, economic, and other descriptive characteristics of the study participants (*N* = 412)^*†*^.

**Variable**	**Total**	**Food secure** ***n* = 236 (57.3%)**	**Food insecure** ***n* = 176 (42.7%)**	***p*-value^‡^**
**Age**	33.80 ± 12.02	33.22 ± 12.45	34.57 ± 11.42	0.262
**Gender**				
Male	92 (22.3%)	50 (21.2%)	42 (23.9%)	0.519
Female	320 (77.7%)	186 (78.8%)	134 (76.1%)	
**Marital status**				
Single/widowed/married	241 (58.5%)	143 (60.6%)	98 (55.7%)	0.317
Married	171 (41.5%)	93 (39.4%)	78 (44.3%)	
**Education level**				
Intermediate and below	9 (2.1%)	1 (0.4%)	8 (4.5%)	0.002
Secondary	20 (4.9%)	7 (3.0%)	13 (7.4%)	
University	383 (93.0%)	228 (96.6%)	155 (88.1%)	
**Employment status**				
Employed	250 (60.7%)	137 (58.1%)	113 (64.2%)	0.206
Unemployed	162 (39.3%)	99 (41.9%)	63 (35.8%)	
**Monthly income**				
Low (<1,000$)	61 (14.8%)	26 (11.0%)	35 (19.9%)	<0.001
Intermediate (1,000$-2000)	104 (25.2%)	45 (19.1%)	59 (33.5%)	
High (>2,000$)	207 (50.2%)	138 (58.5%)	69 (39.2%)	
Refuse to answer	40 (9.7%)	27 (11.4%)	13 (7.4%)	
**Source of income at home is from individuals work**				
Yes	383 (93.0%)	223 (94.5%)	160 (90.9%)	0.103
No	18 (4.4%)	6 (2.5%)	12 (6.8%)	
Refuse to answer	11 (2.7%)	7 (3.0%)	4 (2.3%)	
**Receiving financial support from relatives**				
Yes	60 (14.6%)	26 (11.0%)	34 (19.3%)	0.050
No	342 (83.0%)	205 (86.9%)	137 (77.8%)	
Refuse to answer	10 (2.4%)	5 (2.1%)	5 (2.8%)	
**Receiving financial assistance from governmental or non-governmental institutions?**				
Yes	11 (2.7%)	9 (3.8%)	2 (1.1%)	0.232
No	393 (95.4%)	223 (94.5%)	170 (96.6%)	
Refuse to answer	8 (1.9%)	4 (1.7%)	4 (2.3%)	
**Receiving any income/aid in foreign currency**				
Yes	99 (24.0%)	65 (27.5%)	34 (19.3%)	0.153
No	297 (72.1%)	162 (68.6%)	135 (76.7%)	
Refuse to answer	16 (3.9%)	9 (3.8%)	7 (4.0%)	
**Perceived financial status**				
Poor	73 (17.7%)	19 (8.1%)	54 (30.7%)	<0.001
Average	314 (76.2%)	198 (83.9%)	116 (65.9%)	
Rich	6 (1.5%)	5 (2.1%)	1 (0.6%)	
Refuse to answer	19 (4.6%)	14 (5.9%)	5 (2.8%)	
**Taking medication for insomnia**				
Yes	36 (8.7%)	11 (4.7%)	25 (14.2%)	0.001
No	376 (91.3%)	225 (95.3%)	151 (85.8%)	
**Taking medication for depression**				
Yes	27 (6.6%)	9 (3.8%)	18 (10.2%)	0.009
No	385 (93.4%)	227 (96.2%)	158 (89.8%)	
**Taking medication for anxiety**				
Yes	19 (4.6%)	5 (2.1%)	14 (8.0%)	0.005
No	393 (95.4%)	231 (97.9%)	162 (92.0%)	
**Smoking status**				
Yes	127 (30.8%)	67 (28.4%)	60 (34.1%)	0.215
No	285 (69.2%)	169 (71.6%)	116 (65.9%)	
**Alcohol consumption**				
Yes	188 (45.6%)	116 (49.2%)	72 (40.9%)	0.097
No	224 (54.4%)	120 (50.8%)	104 (59.1%)	
		**Mean ± SD**	**Mean ± SD**	
Fear of poverty	6.50 ± 2.73	5.80 ± 2.78	7.44 ± 2.36	<0.001
Fear of COVID-19 score	14.91 ± 5.98	13.94 ± 5.60	16.22 ± 6.25	<0.001
Household crowding index	1.00 ± 0.48	0.94 ± 0.42	1.09 ± 0.55	0.002
Financial wellbeing scale (IFDFW)	4.31 ± 2.12	5.14 ± 2.09	3.19 ± 1.57	<0.001

† *Continuous variables were presented as means ± standard deviation (SD) and categorical variables were presented as frequencies and percentages n (%)*.

‡ *The chi-square test was used for the comparison between categorical variables whereas the independent-sample t-test was used to compare continuous variables between the food security and insecurity groups. Statistical significance was presented as p < 0.05*.

The mean age of the participants was 33.80 ± 12.02 years; the majority of the participants were females (77.7%) and with a university education level (93.0%). In addition, less than two-thirds of the survey respondents were employed (60.7%), 58.5% were single, and 50.2% reported a high monthly income (>2,000$). Most participants had their work as the source of income (93.0%), did not receive any aid neither from relatives (83.0%) nor from governmental or non-governmental institutions (95.4%). The majority had an average perceived financial status (76.2%), and 72.1% did not receive any aid in foreign currency. Almost all the participants did not report taking any medications for insomnia (91.3%), depression (93.4%), or anxiety (95.4%). The mean fear of poverty was 6.50 ± 2.73, the mean fear of COVID-19 was 14.91 ± 5.98, the mean household crowding index was 1.00 ± 0.48, and the mean financial status was 4.31 ± 2.12.

### Bivariate Analysis

A significantly higher proportion of food-secure participants had a high income (58.5 vs. 39.2%, *p* < 0.001), a university education level (96.6 vs. 88.1%, *p* = 0.002), and an average to rich perceived financial status (86 vs. 66.5%) as compared to those who were food insecure. Those who took medications for insomnia, depression, or anxiety were food insecure as compared to their food secure counterparts. Moreover, a significantly higher mean of fear of poverty, fear of COVID-19, and household crowding index was found in food-insecure vs. food-secure participants. However, a significantly higher financial wellbeing score was found among those who were food secure as compared to food insecure (5.14 vs. 3.19, *p* < 0.001) (Table 1).

Table 2 shows the bivariate analysis taking the two components of quality of life (PCS-SF12 and MCS-SF12) as the dependent variables. The results showed a significantly higher mean of physical QOL (PCS-SF12) among those who were food secure, had a university education level, and consumed alcohol.

**Table 2:** Bivariate associations between food insecurity, sociodemographic and economic characteristics of study participants with their quality of life parameters SF-12 components (PCS-SF12 and MCS-SF12).

	**PCS-SF12^‡^**	***p*-value^†^**	**MCS-SF12^‡^**	***p*-value^†^**
	**Mean ±SD**		**Mean ±SD**	
**Food insecurity status**				
Food secure	55.22 ± 6.10	0.024	36.06 ± 9.51	0.004
Food insecure	53.34 ± 7.38		32.87 ± 8.45	
**Gender**				
Male	54.89 ± 6.24	0.628	36.30 ± 8.68	0.169
Female	54.44 ± 6.76		34.53 ± 9.39	
**Marital status**				
Single/widowed/married	54.84 ± 6.59	0.335	34.51 ± 9.26	0.359
Married	54.09 ± 6.72		35.50 ± 9.26	
**Monthly income**				
No income	48.59 ± 0.01	0.100	35.52 ± 0.01	0.026
Low (<1.500.000 LL)	52.65 ± 7.68		33.03 ± 8.44	
Intermediate (1.500.000–3.000.000 LL)	54.80 ± 7.19		32.57 ± 9.03	
High (>3.000.000 LL)	55.24 ± 5.64		35.82 ± 8.98	
Refuse to answer	52.96 ± 8.24		37.92 ± 11.04	
**Education level**				
Intermediate and below	58.13 ± 2.33	0.011	29.07 ± 9.63	0.279
Secondary	49.27 ± 11.71		37.54 ± 14.45	
University	54.71 ± 6.32		34.88 ± 8.99	
**Employment status**				
Employed	54.70 ± 6.46	0.602	34.45 ± 9.12	0.296
Unemployed	54.30 ± 6.93		35.59 ± 9.46	
**Perceived financial status**				
Poor	52.91 ± 7.98	0.257	32.10 ± 9.99	0.011
Average	54.91 ± 6.23		35.37 ± 9.08	
Rich	54.05 ± 5.23		44.26 ± 5.24	
Refuse to answer	52.95 ± 9.28		31.76 ± 8.34	
**Smoking status**				
Yes	54.43 ± 6.58	0.854	33.79 ± 9.68	0.184
No	54.58 ± 6.68		35.35 ± 9.07	
**Alcohol consumption**				
Yes	55.44 ± 6.87	0.023	34.07 ± 9.11	0.126
No	53.70 ± 6.34		35.69 ± 9.36	
**Taking medication for insomnia**				
Yes	52.44 ± 6.23	0.145	32.33 ± 7.91	0.198
No	54.69 ± 6.66		35.09 ± 9.33	
**Taking medication for depression**				
Yes	52.58 ± 9.01	0.371	29.00 ± 9.42	0.052
No	54.60 ± 6.57		35.09 ± 9.21	
**Taking medication for anxiety**				
Yes	56.34 ± 7.63	0.469	24.07 ± 7.51	0.002
No	54.50 ± 6.63		35.17 ± 9.15	
	**Correlation coefficient**	***p*** **-value**	**Correlation coefficient**	***p*** **-value**
Age	−0.083	0.146	0.037	0.516
Fear of poverty	−0.037	0.524	−0.260	<0.001
Fear of COVID-19 scale	−0.079	0.171	−0.213	<0.001
Household crowding index	−0.117	0.041	−0.066	0.248
Financial wellbeing scale (IFDFW)	0.069	0.228	0.377	<0.001

‡ *MCS, mental component summary; PCS, physical component summary. PCS and MCS range from 0 (lowest level of health) to 100 (highest level of health)*.

† *In order to compare the categorical variable with two groups with the MCS and PCS scales, the independent-sample t-test was used, whereas the ANOVA test was used to compare between three or more means. Pearson correlation test was used to evaluate the association between continuous variables. Statistical significance was presented as p < 0.05*.

A significantly higher mean of mental quality of life (MCS-SF12) was found among those who were food secure, wealthy, had a high income, and did not take any medications for anxiety. In addition, a significantly higher mean of financial wellbeing was associated with higher mental QOL, whereas higher fear of poverty and higher fear of COVID-19 were significantly associated with lower mental QOL.

### Multivariable Analysis

The logistic regression model, taking the food security as the dependent variable, showed that participants with high financial wellbeing were less food insecure (ORa = 0.60; 95%CI: 0.51; 0.71). The fear of poverty, household crowding index, monthly income, education level, employment status and perceived financial status were not related to food security (*p* > 0.05 for all) (Table 3).

**Table 3:** Logistic regression analysis taking the food security/food insecurity as the dependent variable.

	**Beta**	***p*-value**	**ORa**	**95% Confidence interval**	
				**Lower bound**	**Upper bound**
Fear of poverty	0.001	0.978	1.001	0.901	1.113
Financial wellbeing scale	−0.504	<0.001	0.604	0.514	0.711
Household crowding index	0.412	0.110	1.510	0.911	2.504
Monthly income (low vs. no income*)	0.553	0.263	1.739	0.660	4.580
Monthly income (intermediate vs. no income*)	0.566	0.208	1.760	0.730	4.246
Monthly income (high vs. no income*)	0.290	0.503	1.337	0.572	3.124
Education level (Secondary vs. primary*)	−0.990	0.426	0.371	0.032	4.258
Education level (University vs. primary*)	−1.682	0.136	0.186	0.020	1.694
Financial status (Average vs. poor*)	−0.132	0.657	0.876	0.488	1.571
Financial status (Rich vs. poor*)	0.357	0.770	1.429	0.131	15.540
Employment status (employed vs. unemployed*)	0.059	0.819	1.061	0.640	1.759

Variables entered in the model: fear of poverty, Household crowding index, Financial wellbeing scale, monthly income, education level, employment status and financial status. The * symbol indicates the reference groups.

Two major linear regression models were performed, taking the components of QOL as the dependent variables.

When taking the physical QOL (PCS-SF12) as the dependent variable the results showed that in the first model, taking the sociodemographic characteristics as the independent variables, a higher household crowding index (Beta = −2.07, 95%CI: −3.77; −0.37) and a secondary level of education (Beta = −9.76, 95% CI: −17.23; −2.28) were significantly associated with a lower physical quality of life (lower PCS-SF12) (Table 4, Model 1). When adding the food security variable to the models, the results showed that food insecurity tended to significance with physical quality of life (Beta = −1.48, 95% CI: −3.10; 0.13). In the third model, the financial wellbeing and lifestyle variables were added; the results showed that higher household crowding index (Beta = −1.80, 95% CI: −3.53; −0.07) and secondary education level (Beta = -9.88, 95% CI: −17.37; −2.38) were related to a lower physical QOL. However, financial wellbeing and lifestyle variables were not significant (*p* > 0.05 for all) (Table 4, model 3).

**Table 4:** Multivariable analysis exploring associations between food insecurity, financial wellbeing sociodemographic and economic characteristics of study participants with their quality of life parameters [SF-12 components (PCS-SF12 and MCS-SF12)].

	**PCS-SF12 total scale**			**MCS-SF12 total scale**		
	**UB (95% CI)**	**SB**	***p*-value**	**UB (95% CI)**	**SB**	***p*-value**
**Model 1** ^†^						
Gender	−0.28 (−2.14; 1.56)	−0.01	0.759	−1.25 (−3.79; 1.28)	−0.05	0.332
Age	−0.06 (−0.13; 0.01)	−0.10	0.076	0.03 (−0.06; 0.13)	0.04	0.481
Household crowding index	−2.07 (−3.77;−0.37)	−0.14	0.017	−0.42 (−2.75; 1.90)	−0.02	0.721
Education level (secondary vs. primary^*^)	−9.76 (−17.23;−2.28)	−0.28	0.011	5.89 (−4.32; 16.11)	0.12	0.258
Education level (university vs. primary^*^)	−5.49 (−12.15; 1.16)	−0.18	0.106	1.01 (−8.09; 10.12)	0.02	0.826
Financial status (Average vs. poor^*^)	1.20 (−0.81; 3.21)	0.07	0.242	2.80 (0.05; 5.56)	0.12	0.046
Financial status (Rich vs. poor^*^)	−1.01 (−7.17; 5.14)	−0.02	0.745	9.34 (0.91; 17.76)	0.12	0.030
Fear of poverty	−0.04 (−0.32; 0.23)	−0.02	0.753	−0.72 (−1.09;−0.33)	−0.22	<0.001
Monthly income (low vs. no income^*^)	−0.02 (−3.17; 3.13)	−0.01	0.989	−2.64 (−6.96; 1.67)	−0.09	0.229
Monthly income (intermediate vs. no income^*^)	2.54 (−0.30; 5.38)	0.16	0.080	−3.51 (−7.40; 0.37)	−0.16	0.076
Monthly income (high vs. no income^*^)	2.33 (−0.18; 4.84)	0.17	0.069	−2.09 (−5.53; 1.34)	−0.11	0.231
**Model 2** ^†^						
Food insecurity vs. food security^*^	−1.48 (−3.10; 0.13)	−0.10	0.072	−1.46 (−3.68; 0.75)	−0.07	0.195
Gender	−0.31 (−2.16; 1.53)	−0.02	0.738	−1.27 (−3.81; 1.25)	−0.05	0.322
Age	−0.06 (−0.13; 0.01)	−0.10	0.092	0.04 (−0.06; 0.14)	0.05	0.440
Household crowding index	−1.99 (−3.68;−0.29)	−0.13	0.022	−0.34 (−2.67; 1.98)	−0.02	0.771
Education level (secondary vs. primary^*^)	−9.78 (−17.23;−2.34)	−0.28	0.010	5.86 (−4.34; 16.06)	0.12	0.259
Education level (university vs. primary^*^)	−5.65 (−12.29; 0.97)	−0.19	0.094	0.85 (−8.25; 9.95)	0.02	0.854
Financial status (Average vs. poor^*^)	0.97 (−1.05; 2.99)	0.06	0.346	2.57 (−0.19; 5.35)	0.11	0.069
Financial status (Rich vs. poor^*^)	−1.54 (−7.70; 4.62)	−0.03	0.622	8.82 (0.37; 17.27)	0.12	0.041
Fear of poverty	0.01 (−0.27; 0.29)	0.01	0.928	−0.66 (−1.05;−0.27)	−0.20	0.001
Monthly income (low vs. no income^*^)	0.19 (−2.96; 3.34)	0.01	0.905	−2.43 (−6.76; 1.89)	−0.09	0.269
Monthly income (intermediate vs. no income^*^)	2.67 (−0.16; 5.51)	0.17	0.064	−3.38 (−7.27; 0.50)	−0.15	0.088
Monthly income (high vs. no income^*^)	2.38 (−0.11; 4.89)	0.18	0.062	−2.04 (−5.47; 1.39)	−0.11	0.243
**Model 3** ^†^						
Gender	0.05 (−1.94; 2.05)	0.003	0.958	−2.79 (−5.37;−0.22)	−0.12	0.034
Age	−0.06 (−0.13; 0.02)	−0.10	0.120	0.10 (0.01; 0.20)	0.13	0.028
Household crowding index	−1.80 (−3.53;−0.07)	−0.12	0.041	−0.26 (−2.49; 1.97)	−0.01	0.819
Education level (secondary vs. primary^*^)	−9.88 (−17.37;−2.38)	−0.28	0.010	5.03 (−4.62; 14.69)	0.11	0.306
Education level (university vs. primary^*^)	−5.79 (−12.47; 0.88)	−0.19	0.089	1.05 (−7.56; 9.66)	0.02	0.810
Financial status (Average vs. poor^*^)	1.14 (−0.92; 3.22)	0.07	0.277	0.90 (−1.76; 3.57)	0.04	0.506
Financial status (Rich vs. poor^*^)	−1.30 (−7.61; 5.00)	−0.02	0.685	6.51 (−1.62; 14.64)	0.09	0.116
Fear of poverty	−0.05 (−0.40; 0.29)	−0.02	0.777	0.05 (−0.39; 0.50)	0.02	0.798
Monthly income (low vs. no income^*^)	0.07 (−3.12; 3.28)	0.004	0.961	−0.65 (−4.79; 3.47)	−0.02	0.754
Monthly income (intermediate vs. no income^*^)	2.50 (−0.36; 5.38)	0.16	0.087	−2.36 (−6.07; 1.34)	−0.11	0.210
Monthly income (high vs. no income^*^)	2.25 (−0.26; 4.77)	0.17	0.080	−1.72 (−4.97; 1.53)	−0.09	0.299
Food insecurity vs. food security^*^	−1.52 (−3.22; 0.17)	−0.11	0.078	0.21 (−1.97; 2.39)	0.01	0.851
Financial wellbeing scale (IFDFW)	−0.15 (−0.68; 0.37)	−0.05	0.574	1.63 (0.94; 2.31)	0.36	<0.001
Fear of COVID-19	−0.03 (−0.16; 0.09)	−0.03	0.603	−0.21 (−0.37;−0.04)	−0.14	0.012
Alcohol consumption	−0.86 (−2.33; 0.60)	−0.07	0.247	0.75 (−1.14; 2.64)	0.04	0.435
Smoking status (smoker vs. non-smoker^*^)	−0.29 (−1.70; 1.11)	−0.03	0.682	2.82 (1.00; 4.64)	0.18	0.002

*Variables entered in model 1: Gender, age, household crowding index, education level, financial status, fear of poverty, monthly income and household crowding index. Variables entered in model 2: Gender, age, household crowding index, education level, financial status, fear of poverty, monthly income and household crowding index and food insecurity. Variables entered in model 3: Gender, age, household crowding index, education level, financial status, fear of poverty, monthly income, household crowding index, food insecurity, financial wellbeing, fear of COVID-19, alcohol consumption and smoking status*.

† *Model 1 explores the relationship between sociodemographic variables and quality of life (QOL) parameters (PCS-SF12 and MCS-SF12)*.

† *Model 2 explores the relationship between food security variable with QOL parameters, adjusting for sociodemographic variables, including Gender, age, household crowding index, education level, financial status, fear of poverty, monthly income and household crowding index*.

† *Model 3 explores the relationship between food security, lifestyle variables and financial wellbeing with QOL parameters adjusting for sociodemographic variables (Gender, age, household crowding index, education level, financial status, fear of poverty, monthly income and household crowding index) and fear of COVID-19. UB, Unstandardized adjusted regression coefficients; SB, Standardized adjusted regression coefficients. All results in the models were presented as adjusted beta-coefficients with 95%CI*.

* *Reference group*.

When considering the mental quality of life as the dependent variable, the results in the first model showed that an average (Beta = 2.80, 95% CI: 0.05; 5.56) and rich (Beta = 9.34, 95% CI: 0.91; 17.76) financial status were associated with higher mental QOL. However, higher fear of poverty (Beta = −0.72, 95% CI: −1.09; −0.33) was associated with lower mental QOL. When adding the food security variable to the second model, the results showed no significant association between food insecurity and mental QOL (Beta = − 1.46, 95% CI: −3.68; 0.75). In the third model, by adding the financial wellbeing and lifestyle variables; the results showed that higher financial wellbeing (Beta = 1.63, 95% CI: 0.94; 2.31), higher age (Beta = 0.10, 95% CI: 0.01; 0.20) and being a smoker (Beta = 2.82, 95% CI: 1.00; 4.64) were related to a higher mental QOL. However, higher fear of COVID-19 (Beta = −0.21; 95% CI: −0.37; −0.04) was significantly associated with lower mental QOL (Table 4, model 3).

### Mediation Analysis

**Figure 2:**
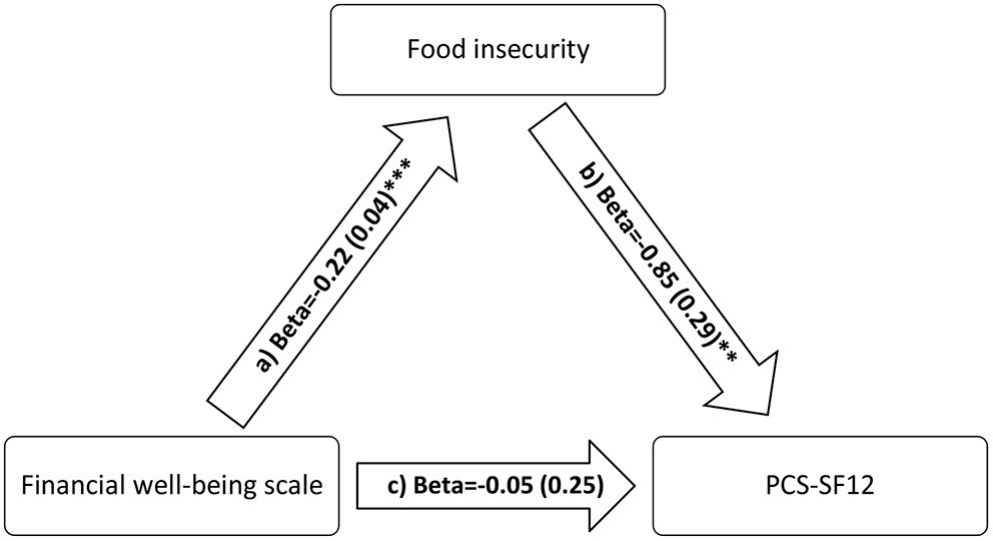
a) Relation between financial wellbeing scale and food insecurity (*R*^2^ = 19.16%); b) Relation between food insecurity and PCS-SF12 (*R*^2^ = 5.68%); c) Relation between financial wellbeing and PCS-SF12 (*R*^2^ = 3.01%). Numbers are displayed as regression coefficients (standard error). **p < 0.01; ***p < 0.001.

**Figure 3:**
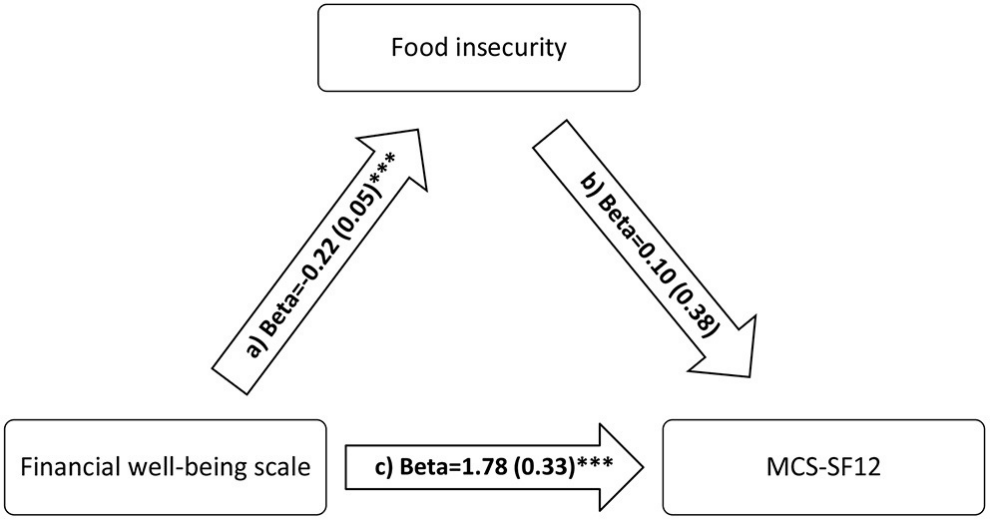
a) Relation between financial wellbeing scale and food insecurity (*R*^2^ = 19.16%); b) Relation between food insecurity and MCS-SF12 (*R*^2^ = 16.57%); c) Relation between financial wellbeing and MCS-SF12 (*R*^2^ = 16.55%). Numbers are displayed as regression coefficients (standard error). ***p < 0.001.

The FI variable mediated the association between financial wellbeing and physical QOL (Table 5; Figure 2) but not mental QOL (Table 5; Figure 3).

**Table 5:** Mediation analyses.

**Model 1: taking the financial wellbeing as independent variables, food insecurity as mediators and PCS-SF12 as the dependent variable**.						
	**Direct effect**			**Indirect effect**		
	**Beta**	**SE**	***p-value***	**Beta**	**Boot SE**	**Boot CI**
Financial wellbeing scale (IFDFW) on PCS-SF12	−0.14	0.26	0.575	0.19	0.08	0.02; 0.36^*^
**Model 2: taking the financial wellbeing as independent variables, food insecurity as mediators as mediators and MCS-SF12 as the dependent variable**.						
	**Direct effect**			**Indirect effect**		
	**Beta**	**SE**	***p-value***	**Beta**	**Boot SE**	**Boot CI**
Financial wellbeing scale (IFDFW) on MCS-SF12	1.80	0.34	<0.001	−0.02	0.08	−0.20; 0.14

* *Indicates significant mediation*.

## Discussion

To the best of our knowledge, this study is the first to explore the relationship between food insecurity, financial wellbeing, and quality of life parameters in Lebanon. It is known that financial constraint is related to FI (22) and is an indicator of the quality of life and wellbeing (23). In turn, FI was found in literature to be related to poor mental (13, 14) and physical health (11, 12). However, there is an absence of a similar framework, exploring the relationships between these factors (financial wellbeing, FI and QOL) in the literature.

Results showed that about 43% of study participants were experiencing FI, with 31% being mildly food insecure, 10% moderately food insecure, and 1.5% severely food insecure. The prevalence of FI in the present study was slightly lower than that in a previous study conducted in Lebanon from nationally representative samples of 1,133 Lebanese participants, where 53% of the Lebanese households had a poor food consumption score (19). In 2019, a study found that food insecurity was 49.3% among Lebanese children aged 4–18 years (30), while data from 2014 reported a prevalence of 42% among vulnerable Lebanese and Palestinian refugees in South Lebanon (31), higher than FI rates collected by the Gallup World Poll data in Lebanon between 2015 and 2017 showing that 11.7–15.3% of the Lebanese households experienced some form of FI (21). The alarming FI rates reported in our study were also in line with recently published data from humanitarian and international agencies (32–34) working closely with the Lebanese and refugee populations in the country, showcasing the catastrophic effect of the multiple crises that Lebanon has been undergoing since 2019 ranking it among the top three crises since mid-nineteenth century worldwide (3). In parallel, the country has undergone an unprecedented financial collapse with a devaluation in the currency value (plummeting from 1 USD/1500 LBP conversion rate to 1 USD/25,000 LBP rate). Moreover, banks tightened limits on foreign currency, cash withdrawals from individual accounts were stopped, and a limit was set on local currency. Consequently, the price of the basic food basket has increased to reach 483% in January 2022 (4), further exacerbating the food security status of the Lebanese households.

Consistent with the literature, study findings showed that food security was significantly associated with higher income, a university education level, a lower crowding index, and higher financial wellbeing. Previous studies conducted in Lebanon revealed that low levels of education, unemployment, low income, and higher crowding were significant correlates of household FI (30, 35). A study has found that higher education level affects food security after controlling for the household wealth index (36). Financial and economic crises have also been shown to have the highest impact on the food security status in developing countries (37), with economic status correlating directly with FI (38).

Bivariate analyses showed that food-secure individuals had significantly higher physical and mental QOL scores compared to their food insecure counterparts. FI was significantly higher among those who take medications for insomnia, depression, or anxiety, and food-insecure participants had a significantly higher mean of fear of poverty and fear of COVID-19. Those results are aligned with what has been found in the literature, and the effect of FI on mental health is elicited through deep psychological stress due to the negative psychological and behavioral experiences leading to mental health problems among youth and adults and can be associated with depressive symptoms among those struggling with FI (39–42). FI is also related to poor general physical health (11, 12), where people from food-insecure households perceived their health as poor/fair and scored lower on the physical and mental health components of the SF-12 (43). Food security was associated with better health-related quality of life in several studies conducted among children and adults (44, 45).

Multivariate analyses showed that FI was not significantly associated with lower physical and mental QOL after adjusting for demographic and socioeconomic correlates and the financial wellbeing variable. One of the challenges in isolating the influence of FI on mental health is addressing the effect of financial resources on both mental health and FI (38). For this reason, the mediation analysis was conducted, and the results showed that FI may have mediated the association between financial wellbeing and physical, but not mental QOL. Thus, financial wellness had a direct effect on the mental QOL in Lebanon (away from the path of FI), while its effect on physical QOL is indirect and mediated, at least partially, through FI. In other words, people with a low mental QOL are directly affected by financial constraints and may seem more worried about their overall situation, away from immediate food security concerns. Oppositely, people with lower physical QOL seem to be directly affected by food insecurity, related itself to financial wellness. The direct relationship between FI and physical QOL can be explained by the physiological and biological mechanisms related to feelings of hunger and deprivation that can affect the different physical health domains, including physical functioning, bodily pain, general health, and vitality (42, 46). In addition, the limited or uncertain access to adequate food due to financial limitations can affect the physical QOL by not meeting the nutritional requirements for energy (total calories) from macronutrients, including proteins, carbohydrates, and fats, along with poor intake of essential vitamins and minerals, which may, in turn, affect the general physical health.

In this study, financial wellness was shown to have a direct effect on mental QOL, independent of the FI pathway. A probable explanation could be the direct relationship between financial wellbeing and reduced stress and anxiety levels due to feelings of job security, thus leading to better general mental health and wellbeing (16). Higher financial resources and capabilities can also be associated with the increased ability to access psychosocial and mental health services (MPHSS) (47), which may further explain the positive and strong association between increased financial wellbeing and higher scores on the mental QOL measure in this study, irrespective of the food security status. Nevertheless, further studies are needed to elucidate the associations found between FI, financial wellbeing, and various measures of QOL and wellbeing, particularly in conflict-affected and crisis settings.

Worth noting that the Lebanese have been struggling with compromised mental health due to the long-term effect of exposure to wars, ongoing turmoil, added to social, political, and environmental stressors, including the most recent COVID-19 pandemic and the tragic Beirut port blast, which have taken a toll on the mental and physical health status of the Lebanese population (48–50). Other challenges that may adversely impact the overall mental health and wellbeing of the Lebanese are the persistent social stigmas and cultural taboos related to mental health problems, thus preventing individuals who are in need from seeking access to existing MHPSS services (51). In the past 2 years, the limited financial capacity of the Lebanese population, insufficient subsidies, and the shortage of essential goods and services, including medications, have rendered access to any of these services even further problematic. Moreover, the Lebanese healthcare system has been strained by years of underfunding and ever-increasing demands for serving the Lebanese residents and the high numbers of refugees in the country, including Syrian, Iraqi, and Palestinians (52). All these factors highlight the need for further studies and interventions that tackle the multiple dimensions of health and wellbeing and the gravity of the current situation.

### Strengths and Limitations

To our knowledge, this study is the first to explore the mediating effect of food insecurity on the association between financial wellbeing and QOL. Other strengths of the study include the rigorous methodology through the use of several validated tools. Nevertheless, several limitations should be considered. Causality cannot be established between financial wellbeing, FI, and QOL measures due to the cross-sectional design of the study. The online survey used for data collection might have limited the ability to reach individuals who may be the most vulnerable to FI, and thus results may not be generalizable to the entire Lebanese population. Future studies are necessary to further explore the associations between FI, financial wellbeing, and QOL parameters.

## Conclusion

Food insecurity was prevalent in our study sample. Food-insecure participants reported greater fear of poverty, fear of COVID-19, and FI repercussions on their health and lives.

FI also mediated the association between financial wellbeing and physical, but not mental, QOL parameters. These associations require further exploration in future studies and programs. Our study findings also call for evidence-based policies and interventions to help improve the food security, mental health and overall wellbeing of Lebanese households amidst these unprecedented circumstances.

## Data Availability Statement

The original contributions presented in the study are included in the article/supplementary material, further inquiries can be directed to the corresponding author.

## Ethics Statement

The studies involving human participants were reviewed and approved by the Modern University of Business and Science Ethics Committee (approval reference MU-20211005-26, Oct 2021). The patients/participants provided their written informed consent to participate in this study.

## Author Contributions

JK and PS conceptualized the research design. LJ, HS, and MS provided support in designing the study. JK sought after ethics approval. PS and CH conducted data analysis. JK and CH wrote the original draft of the manuscript. PS, LJ, HS, and MS critically reviewed the manuscript. HS reviewed and edited the manuscript. All authors contributed in the spread of the survey and read and agreed to the published version of the manuscript.

## Conflict of Interest

The authors declare that the research was conducted in the absence of any commercial or financial relationships that could be construed as a potential conflict of interest.

## Publisher's Note

All claims expressed in this article are solely those of the authors and do not necessarily represent those of their affiliated organizations, or those of the publisher, the editors and the reviewers. Any product that may be evaluated in this article, or claim that may be made by its manufacturer, is not guaranteed or endorsed by the publisher.
