# Erratum: Financial wellbeing and quality of life among a sample of the Lebanese population: The mediating effect of food insecurity

**DOI:** 10.3389/fnut.2022.1029025

**Published:** 2022-09-21

**Authors:** Frontiers Production Office

**Affiliations:** Frontiers Media SA, Lausanne, Switzerland

**Keywords:** food insecurity, quality of life, financial wellbeing, Lebanon, physical health, mental health

Due to a production error, there was an error regarding the affiliation for “Hala Sacre.” The author should only have the affiliation “Nutrition Department, Institut National de Santé Publique, d'Épidémiologie Clinique et de Toxicologie (INSPECT-LB), Beirut, Lebanon.”

Due to a production error, there was an error regarding the affiliation for “Pascale Salameh.” The author should have the affiliations, “Nutrition Department, Institut National de Santé Publique, d'Épidémiologie Clinique et de Toxicologie (INSPECT-LB), Beirut, Lebanon,” “Department of Primary Care and Population Health, University of Nicosia Medical School, Nicosia, Cyprus,” “School of Medicine, Lebanese American University, Byblos, Lebanon,” and “Faculty of Pharmacy, Lebanese University, Hadat, Lebanon.”

The publisher apologizes for this mistake. The original version of this article has been updated.

